# High-Power Fiber Laser Welding of High-Strength AA7075-T6 Aluminum Alloy Welds for Mechanical Properties Research

**DOI:** 10.3390/ma14247498

**Published:** 2021-12-07

**Authors:** Abdel-Monem El-Batahgy, Olga Klimova-Korsmik, Aleksandr Akhmetov, Gleb Turichin

**Affiliations:** 1 Manufacturing Technology Department, Central Metallurgical Research and Development Institute (CMRDI), Cairo 11421, Egypt; 2 World-Class Research Center Advanced Digital Technologies, St. Petersburg State Marine Technical University, St. Petersburg 190121, Russia; o.klimova@ltc.ru (O.K.-K.); gleb@ltc.ru (G.T.); 3 Department of Welding and Laser Technologies, Peter the Great St. Petersburg Polytechnic University, St. Petersburg 195251, Russia

**Keywords:** high strength AA7075-T6, high power fiber laser autogenous welding, heat input, microstructure, internal micro-defects, mechanical properties, mechanical mismatching

## Abstract

The results disclosed that both the microstructure and mechanical properties of AA7075-T6 laser welds are considerably influenced by the heat input. In comparison with high heat input (arc welding), a smaller weld fusion zone with a finer dendrite arm spacing, limited loss of alloying elements, less intergranular segregation, and reduced residual tensile stress was obtained using low heat input. This resulted in a lower tendency of porosity and hot cracking, which improved the welded metal’s soundness. Subsequently, higher hardness as well as higher tensile strength for the welded joint was obtained with lower heat input. A welded joint with better mechanical properties and less mechanical discrepancy is important for better productivity. The implemented high-power fiber laser has enabled the production of a low heat input welded joint using a high welding speed, which is of considerable importance for minimizing not only the fusion zone size but also the deterioration of its properties. In other words, high-power fiber laser welding is a viable solution for recovering the mechanical properties of the high-strength AA 7075-T6 welds. These results are encouraging to build upon for further improvement of the mechanical properties to be comparable with the base metal.

## 1. Introduction

Aluminum alloys constitute a fairly representative class of lightweight materials for a wide range of important applications including the transportation sector, where much better fuel efficiency and lower CO_2_ emissions are demanded. Medium strength 5000 and 6000 series alloys are typically used in car bodies due to their good formability, corrosion resistance, and weldability [1,2,3,4]. High-strength 7000 series alloys with their high specific strength, high fracture toughness, good fatigue strength, good formability, and machinability over other Al alloys are used in rather critical applications such as aerospace and aircraft structures [5,6]. The growing demand for better performance and improved safety vehicles has extended the use of the high-strength AA7075 to the automotive industry, including high-speed railway vehicles [7,8,9]. The good balance of its properties, with tensile strength over 500 MPa, is achieved through the formation of strengthening precipitates [10,11,12].

As a part of the fabrication process, welding is one of the most important manufacturing technologies used in the aluminum alloy industry. The high conductivity, high reflectivity, high reactivity, and high coefficient of thermal expansion make welding aluminum alloys difficult [13,14]. Over decades, conventional fusion welding processes are widely used for joining aluminum alloys. These processes allow obtaining acceptable mechanical properties with minimum distortion of welded structures [15,16,17,18]. However, welding of the high-strength 7075 aluminum alloy is a great challenge due to its high thermal sensitivity and low eutectic liquidus temperature range. It is considered not weldable using the conventional fusion welding processes [19,20]. Mechanical fastening, such as riveting, has been a common method of jointing this high strength aluminum alloy. The many of rivets employed in the design considerably increase the mass of the structure, along with the additional complexities associated with stress accumulation and corrosion [21,22]. The problems with welding and rivet joining of this high-strength Al alloy changed dramatically with the invention of the solid-state friction stir welding (FSW) process. Currently, FSW is the best choice for the much better quality of the high-strength aluminum welded structures in aerospace and aircraft industries [23,24,25]. However, the FSW technique with its non-flexibility and low welding speed is an issue concerning adaptability/applicability in the automotive industry where high productivity is demanded. Nowadays, resistance spot welding (RSW) is an efficient and reliable technique applied in this industry where both the quality and productivity of steel vehicles are satisfied [26,27]. Unfortunately, the RSW technique is not the preferred choice for aluminum alloys vehicles due to the much higher electrical and thermal conductivity of the aluminum alloys [28,29].

Appropriate high-strength consumables for high-strength Al alloys are, unfortunately, not yet commercially available. Hence, autogenous electron beam welding (EBW) and laser beam welding (LBW) are extremely attractive processes for a better weld quality of these alloys [30,31,32,33]. For both processes, sound welds with a high depth–width ratio, minimal distortion, and a high welding speed are obtained. EBW is restricted by conducting the welding in a vacuum chamber. Nowadays, using LBW in manufacturing steel car bodies as well as medium-strength Al alloys car bodies is well established in the automotive industry [34,35]. In particular, solid-state lasers are more attractive due to their higher flexibility with using fiber-optic delivery systems. Recently, investigations on LBW of the high-strength 7075 Al alloy for automotive applications are being pursed [22]. Hot cracking of its fusion welded joints and softening of the heat-affected zone (HAZ) are the main problems that remarkably deteriorate its mechanical properties [36,37]. The recently developed high-power solid-state fiber laser with its much better beam quality than previous conventional lasers opens the way for expanding the high-strength 7075 Al alloy sheets to the automotive industry for higher-performance vehicles. This research zone is far from ending, and a lot of research studies are necessary to carry out for deep understanding and minimizing of the welding problems of this alloy. The present research work aims at investigating the feasibility of recovering the mechanical properties of the high-strength AA7075-T6 sheets using a high-power fiber laser process.

## 2. Materials and Methods

**Figure 1:**
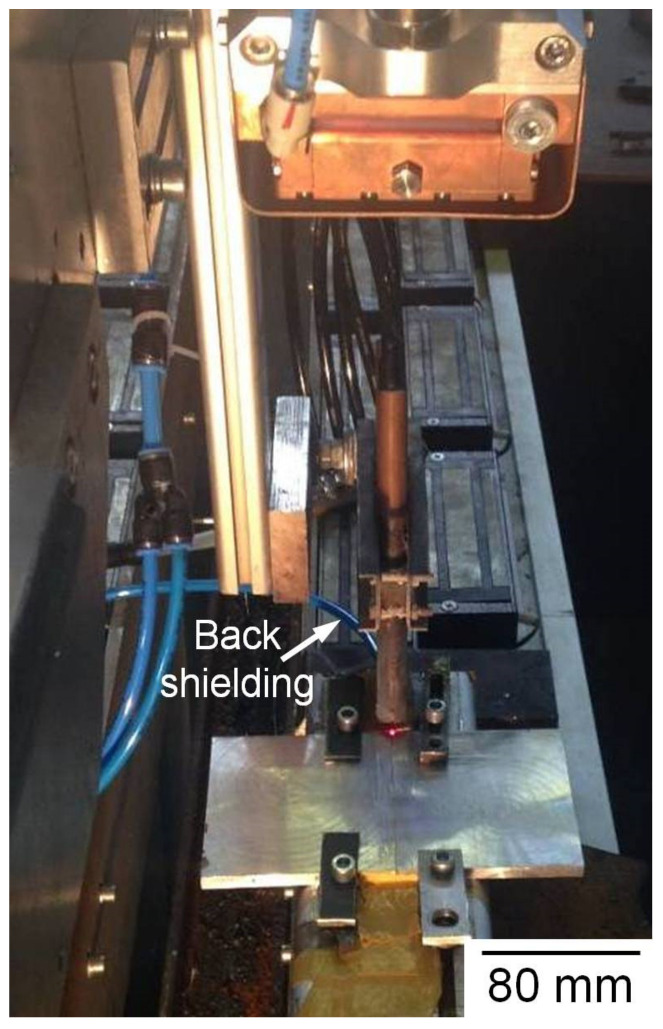
Experimental setup of laser beam welding.

The used materials are 6 mm thick commercial high-strength AA7075-T6 sheets. Their chemical composition and mechanical properties are presented at Table 1. The average measured mechanical properties were 521 MPa tensile strength and 189 HV hardness. Weld samples with 100 × 100 × 6 mm^3^ sizes were prepped for laser beam welding tests. Preliminary surface treatment of the sheets was done by using a stainless steel bristle, which was followed by light polishing with 400 grit SiC paper and defatting with acetone. A photograph of the experimental setup of laser beam welding is shown in Figure 1, where the weld samples were kept strongly in place with a fixture to prevent deformation. Both bead-on-plate and single pass autogeneous square butt joints were made using a fiber laser system with a maximum output power of 16 kW. The direction of welding was perpendicular to the rolling direction. The laser beam was focused to a 0.5 mm spot size using a 500 mm focal length lens and 4 mm nozzle diameter. In order to minimize the beam reflection that prevents the damage of optics, the laser head was inclined 15° from the vertical position. The laser welding parameters investigated such as laser power (P), welding speed (S), defocusing distance (Dd), and flow rate of shielding gas are shown in Table 2. The laser power was varied between 5.0 and 9.0 kW, the welding speed was changed between 2.0 and 7.8 m/min, the defocusing distance was varied between −10.0 and +10.0 mm, while the weld pool top and bottom shielding was performed with argon gas at a flow rate between 15 and 25 L/min.

**Figure 2:**
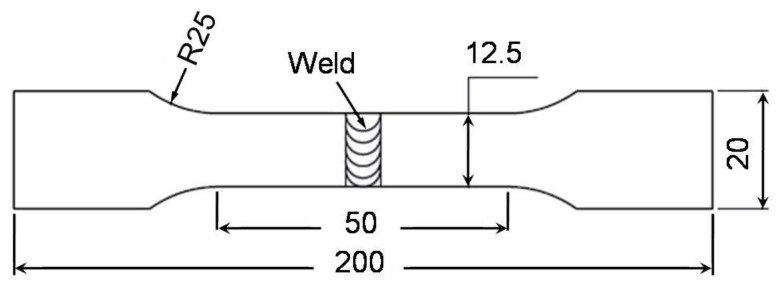
Schematic illustration and dimensions of transverse tensile test specimen of welded joint. Dimensions: mm.

Laser beam welded joints were exposed to visual and X-ray examinations to verify that there were no external and internal welding defects, respectively. Then, metallurgical studies, hardness measures, and tensile tests in the cross-sections of the welds were provided. The structure of the fusion zone was investigated by low-magnification stereoscope (Olympus, Tokyo, Japan), and the microstructure was analyzed with optical and scanning electron microscopes. The Vicker’s microhardness measurements on polished and etched cross-sections were taken at about half the thickness across the welds at a load of 200 gf (0.392 N) for 15 s. Compositional variations across the welds were identified using a scanning electron microscope (SEM) (FEI, Lausanne, Switzerland) equipped with energy-dispersive X-ray spectroscopy (EDS) (Entertek, London, UK) at an accelerating voltage of 20 kV. The tensile strengths of butt welds were assessed at room temperature using tensile samples cut perpendicular to the direction of welding. All tensile tests were performed at a fixed traverse speed of 0.1 mm/min on a 100 kN Shimadzu testing machine (Shimadzu, Kyoto, Japan). Flat tensile samples 50 mm long, 6 mm thick, 12.5 mm wide, and a total length of 200 mm were prepped for the laser beam welds (Figure 2) according to ASME IX. Three specimens were tested in the welded state for each weld parameter as well as for the main material, and the mean value was regarded.

## 3. Results and Discussion

**Figure 3:**
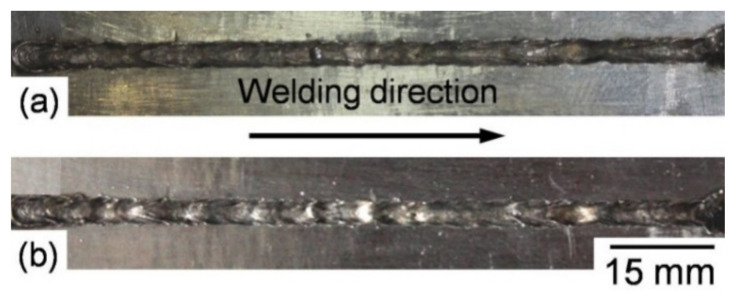
Weld bead surface images of the face side of laser butt-welded joints obtained using the low (**a**) and high (**b**) heat inputs.

The laser beam welding parameters used for producing autogenous square butt-welded joints were defined on the basis of bead-on-plate experiments, which were subject to obtaining a weld bead with full penetration and permissible bead geometry. Two different sets of laser welding parameters were implemented to obtain low and high heat input welded joints (Table 3). The low heat input (90 kJ/m) welded joint was produced using parameters of 9 kW laser power, 6 m/min welding speed, −10 mm defocusing distance, and20 L/min Ar shielding. The high heat input (180 kJ/m) welded joint was obtained using parameters of 6 kW laser power, 2 m/min welding speed, −10 mm defocusing distance, and 20 L/min Ar shielding. Visual inspection of the laser-welded joints produced using the low and high heat inputs showed acceptable weld beads appearance where almost no spatter, no porosities, and no weld bead or crater cracks were observed, as shown from the weld bead surface images in Figure 3. The absence of visual pores is an indication to stability of the keyhole during welding. Radiographic inspection disclosed sound and full penetration welded joints where no unacceptable internal defects were obtained. This is mainly due to a proper selection of the implemented welding conditions for the produced full penetration welds. Radiography-accepted welded joints were subjected to metallurgical and mechanical examinations.

### 3.1. Macrostructure of Laser Beam-Welded Joints

**Figure 4:**
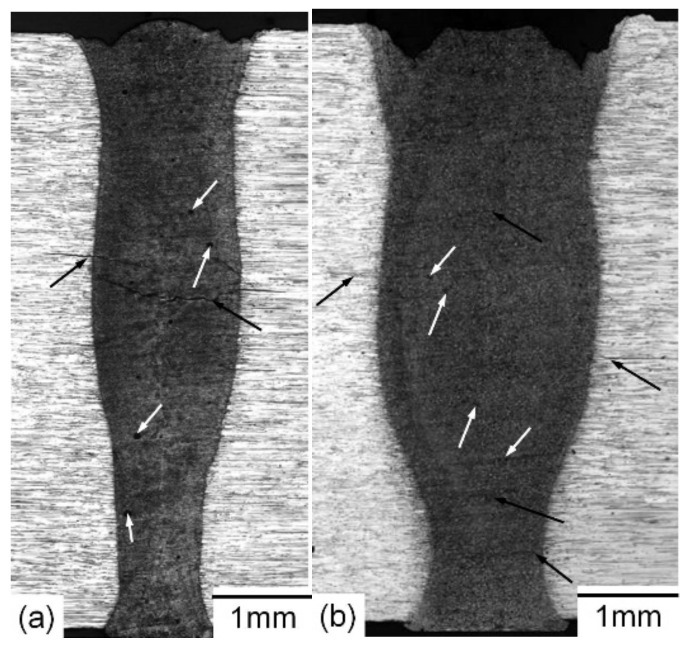
Low-magnification stereoscopic photographs of cross-sections of laser beam-welded joints produced using the low (**a**) and high (**b**) heat inputs. Black arrows indicate micro-cracks, while white arrows indicate micro-porosity.

Low-magnification stereoscopic macrographs of cross-sections of the low and high heat input welds are represented in Figure 4a,b, respectively. It should be noted that the tendency of the laser weld is usually symmetrical relative to the center with typical characteristics of keyhole laser welding. Macroscopic examination confirmed full penetration for both welded joints. The bead of weld parameters depth/relations of 3.5 and 2.3 were derived for the low and high heat input welds, respectively. Otherwise, the size of the fusion zone for the lower heat capacity weld (Figure 4a) is approximately two-thirds that of the high heat input weld (Figure 4b). One of the observed welding defects for both welded joints is the severe undercut that is not acceptable, particularly for critical applications where corrosion and/or stress cycles are concerned. This problem is related mainly to a high degree of metal evaporation during welding, particularly Zn and Mg. The main problem with metal vaporization during welding is the shortage of material in the welding area, which leads to undercutting of the weld. It can be overcome using a filler metal or by implementing hybrid laser welding where filler material addition and work-piece gap tolerance are considered, as has been previously reported [19,33]. On the other hand, indications for internal welding defects including micro-cracking in the weld metal (WM) and partially melted zone (PMZ) as well as micro-porosity in the WM were observed for both joints. Such internal defects are critical and not acceptable, since it negatively affects the welded joint mechanical properties. Then, detailed microscopic examinations were performed.

### 3.2. Microstructure of Laser Beam-Welded Joints

**Figure 5:**
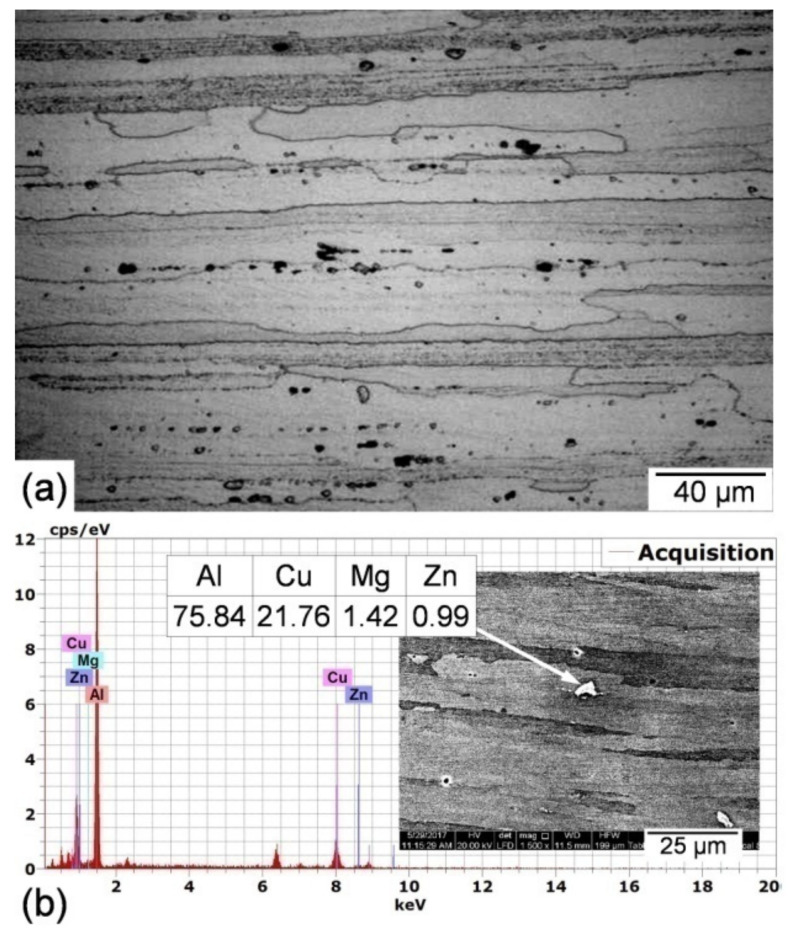
Optical microscopic photograph (**a**) and EDS microanalysis of the strengthening precipitates (**b**) of the base metal.

Optical microscopic photographs and EDS microanalysis of the base metal are shown in Figure 5. The microstructure of the tested AA5075-T6 base metal (BM) is characterized by a coarse, lengthened, band-shaped grain structure with a length of approximately 200 µm in the transverse direction (perpendicular to the centerline of the weld) and a width of 20 µm in the regular direction/all along the thickness and parallel to the centerline of the weld (Figure 5a). This structure is developed by the rolling process during manufacturing of the sheet. Threads of black inclusion particles of 2 to 5 µm in size and irregular form are easily visible. White fine particles dispersed through the matrix were found. EDS microanalysis of these particles (Figure 5b) indicated that they are abundant in Al, Cu, Mg, and Zn, showing the existence of isomorphic phases MgZn and AlCuMg as hardening precipitates in the alloy. An average hardness value of 189 HV was obtained for this microstructure that improves mechanical properties.

**Figure 6:**
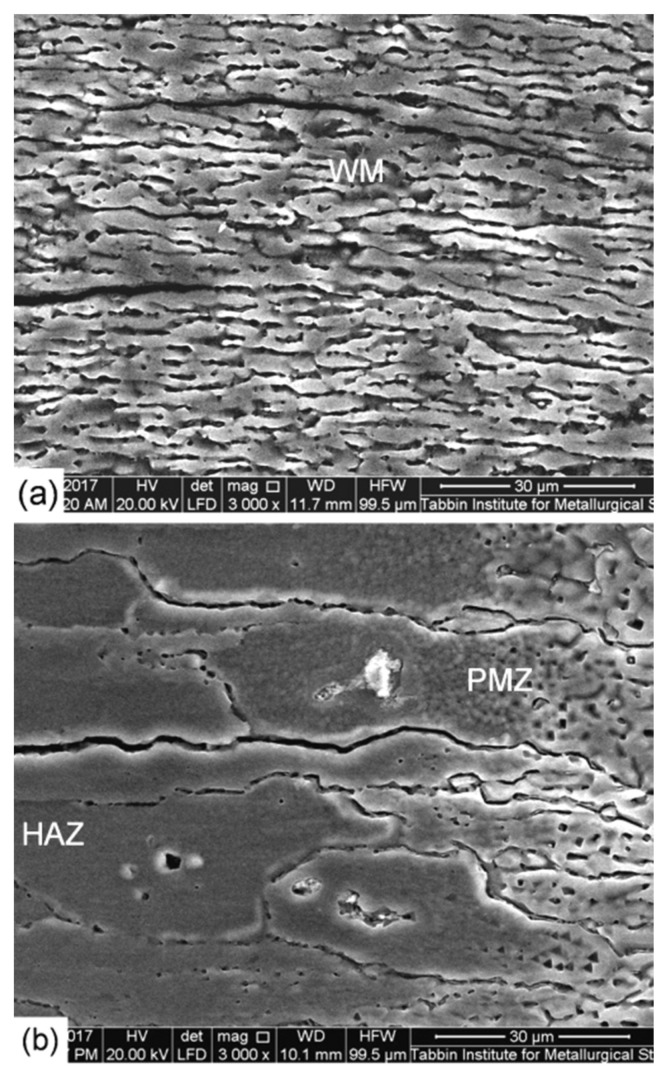
High-magnification SEM photographs of a cross-section (**a**) WM and (**b**) PMZ, HAZ zone of the low heat input laser-welded joint.

**Figure 7:**
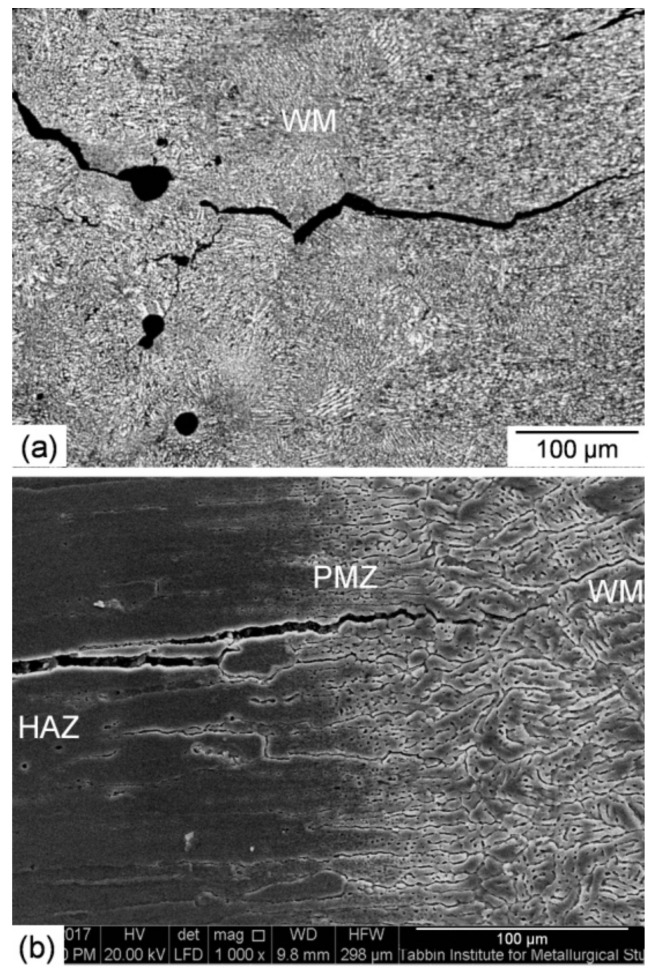
Optical (**a**) and low-magnification SEM (**b**) photographs of a cross-section of the high heat input laser-welded joint.

High-magnification SEM photographs of a cross-section of the low heat input laser beam welded joint are shown in Figure 6. A microscopic study demonstrated that the width of the WM and PMZ is 1.7 mm and 0.25 mm, respectively. It also confirmed the existence of micro-porosity and micro-cracking in the WM (Figure 6a) as well as micro-cracking in the PMZ (Figure 6b). The cracks were initiated and propagated along the dendrite boundaries. Optical and low magnification SEM photographs of a cross-section of the high heat input laser beam welded joint are shown in Figure 7. The width of the WM and PMZ of this joint is 2.3 mm and 0.3 mm, respectively. The most important finding is a higher degree of micro-porosity and micro-cracking in the WM (Figure 7a) as well as micro-cracking in the PMZ (Figure 7b).

The porosity of AA7075-T6 is related mainly to the low vaporization temperature of its alloying elements Zn and Mg, which means a high degree of evaporation during welding. It is affected also by the laser beam mode. The amount of micro-porosity obtained in this study was not relatively severe because the used continuous wave laser beam results in a better keyhole stability compared with a pulsed laser beam mode [38]. The micro-cracking found in both welded joints is a hot cracking that can be classified as solidification cracking in the WM and liquation cracking in the PMZ. This cracking type of the AA7075-T6 welded joint is related mainly to its high thermal expansion coefficient, intense evaporation of its alloying elements Zn and Mg, dissolution of strengthening precipitates, intergranular segregation, and residual tensile stresses. This means that both the porosity and hot cracking tendency is influenced by the heat supplied. Compared to high heat supply, a lower degree of porosity, regarding the size and amount of pores, as well as a considerable reduced tendency of hot cracking were obtained using the low heat input. This is in agreement with other research results [39].

**Figure 8:**
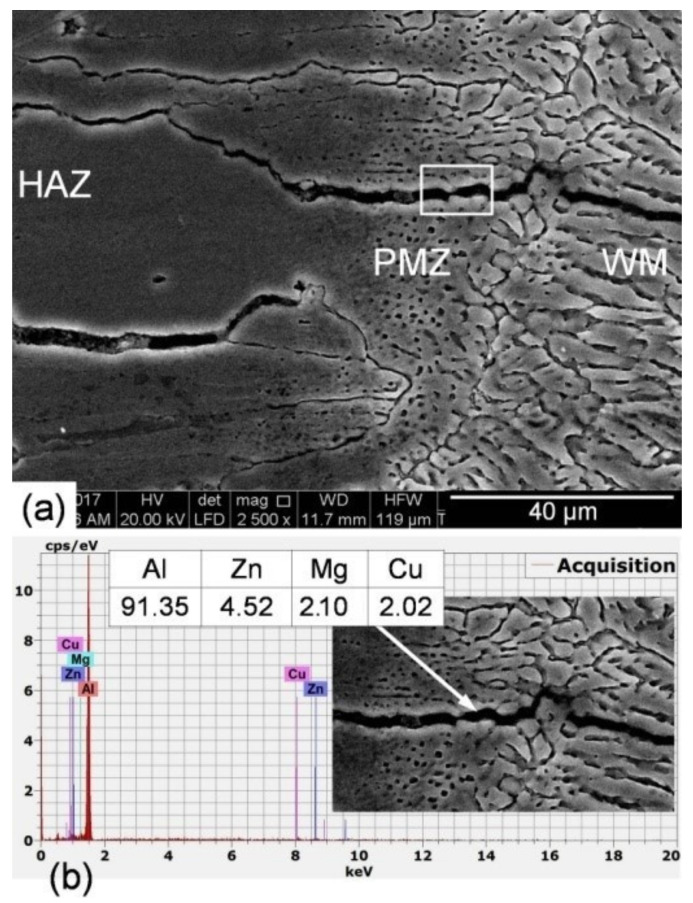
High-magnification SEM photograph of the fusion boundary of the low heat input laser-welded joint (**a**) and typical example of EDS microanalysis of its cracking zone (**b**).

In order to elaborate about the observed internal welding defects’ phenomena, detailed investigations using EDS microanalysis were carried out. High-magnification SEM photographs of the fusion boundary of the low heat input laser-welded joint as well as typical example of EDS microanalysis of its cracking zone are shown in Figure 8. The microstructure of the fusion boundary shows elongated coarse grains in the PMZ and HAZ (Figure 8a). The grains in both zones are slightly larger than those in the used BM. EDS micro-analysis of the PMZ crack (white marked zone in Figure 8a) indicated remarkable elemental loss, particularly Zn and Mg (Figure 8b). Both Zn and Mg, as the core components adding to AA7075, have lower boiling points and much higher vapor pressures than the other alloying components [40]. Gas bubbles could not float up to escape from the surface of the molten pool due to the too short solidification time. Then, bubbles periodically form from the keyhole tip and rise to the surface as they move up the molten pool. Those that are captured on the solidification wall during this process are preserved in the form of porosity. It is believed that porosity adds further contribution to hot cracking formation where porosity will act as stress concentration, sites acceleration, crack initiation, and propagation. Such micro-cracking is not always easily detectable using radiographic testing (RT) and can lead to catastrophic failure while in service due to deteriorated weld properties.

**Figure 9:**
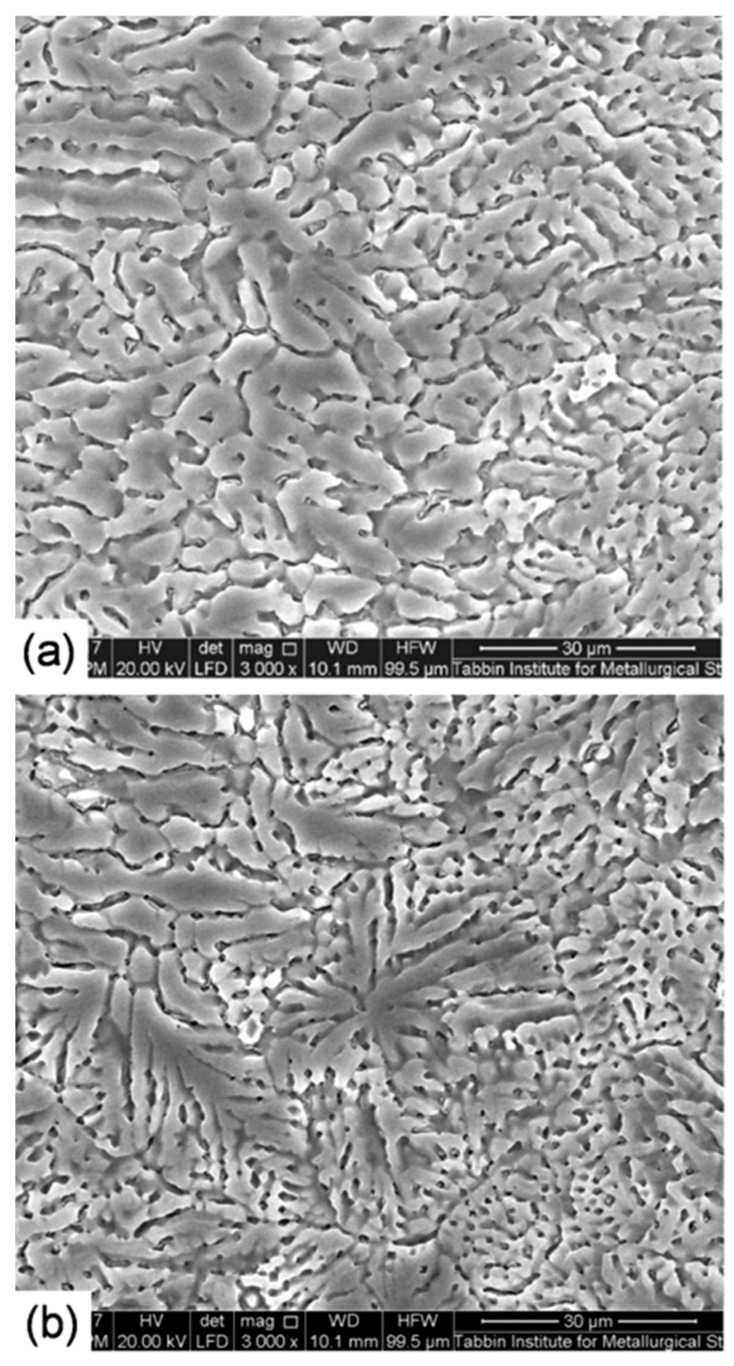
SEM photographs of WM of the high (**a**) and low (**b**) heat input laser-welded joints.

**Figure 10:**
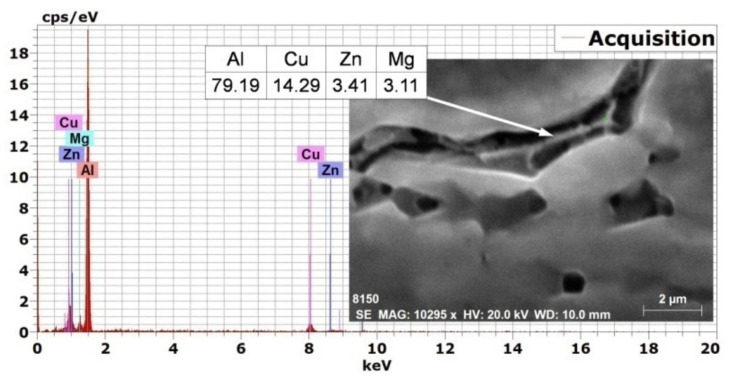
EDS microanalysis of the interdendritic white phase of the high heat input WM.

SEM photographs of the WM of the low and high heat input laser-welded joints are shown in Figure 9. SEM investigation showed a casting metallographical morphology after repeated melting and quick cooling in the process of welding. SEM examinations disclosed a fine dendritic solidification structure for the low heat input WM (Figure 9b), while a coarser dendritic structure was obtained for the high heat input WM (Figure 9a). This type of structures is controlled by the degree of cooling speed as a function of the heat consumption. Faster cooling speed as a result of lower heat consumption is responsible for finer dendrite morphologies. These mean that the laser welding process has radically changed the microstructure of the resulting BM rolled products. The measurement of the solute chemistry within this dendritic structure indicated significant losses of the elements Zn and Mg obtained in the fusion zone due to their lower boiling temperatures than those of the other elements (Figure 10).

### 3.3. Hardness Measurements of Welded Joints

**Figure 11:**
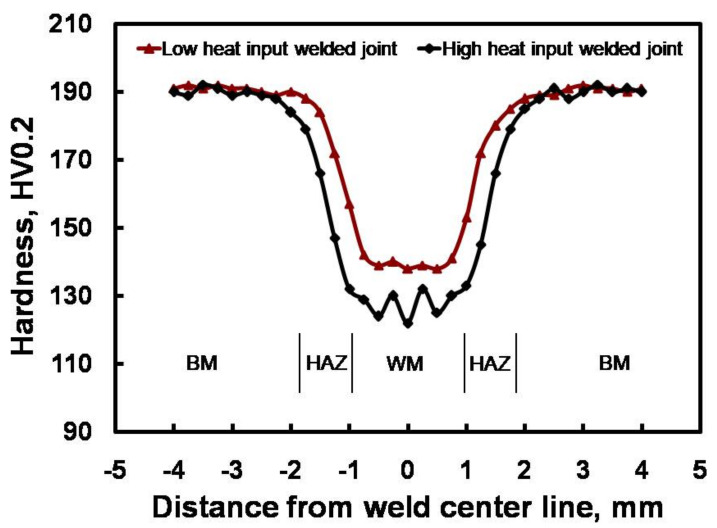
Hardness profiles of cross-sections of the low and high heat input welds.

Since HAZ and WM partially recover their strength through natural aging, both hardness tests and tensile tests were performed 90 days after welding. At this time, the maximum strength due to natural aging was reached. Generally, a 30-day holding period is taken as the lowest time interval before testing aluminum welds. Standard hardness shapes of cross-sections taken from the low and high heat input welded joints are shown in Figure 11. The maximum deviation of the hardness measurements HV0.2 was ±4%. Similar hardness profiles were obtained, and an acceptable weld zone width of 1.7 mm and 2.3 mm was confirmed for the low and high heat input welds, accordingly. HAZ widths of about 1.0 mm and 1.2 mm were obtained for the low and high heat input welds, accordingly. Such a large HAZ width is related mainly to the high heat conductivity coefficient of AA7075. Although the WM microstructure is finer than that of the BM and finer grains can help enhance the weld microhardness, it still could not remedy the low microhardness of the casting pattern. In comparison with the BM (189 HV), the hardness gradually decreases until it reached its minimum values in the WM of both welded joints. The hardness decrease in the HAZ is related to its softening as a result of coarsening and dissolution of strengthening precipitates. The low heat input caused in weld metal with a higher hardness (140 HV) compared to that of the high heat input (125 HV). This is most probably attributed to less degradation for the chemical composition and microstructure of the low heat input WM compared to that of the high heat input. The hardness of the low heat input weld is 74% of the BM, while that of the high heat input is 66%. The weld metal higher hardness of the low heat input welded joint is expected to recover the welded joint tensile strength.

### 3.4. Micro-Chemical Analysis

**Figure 12:**
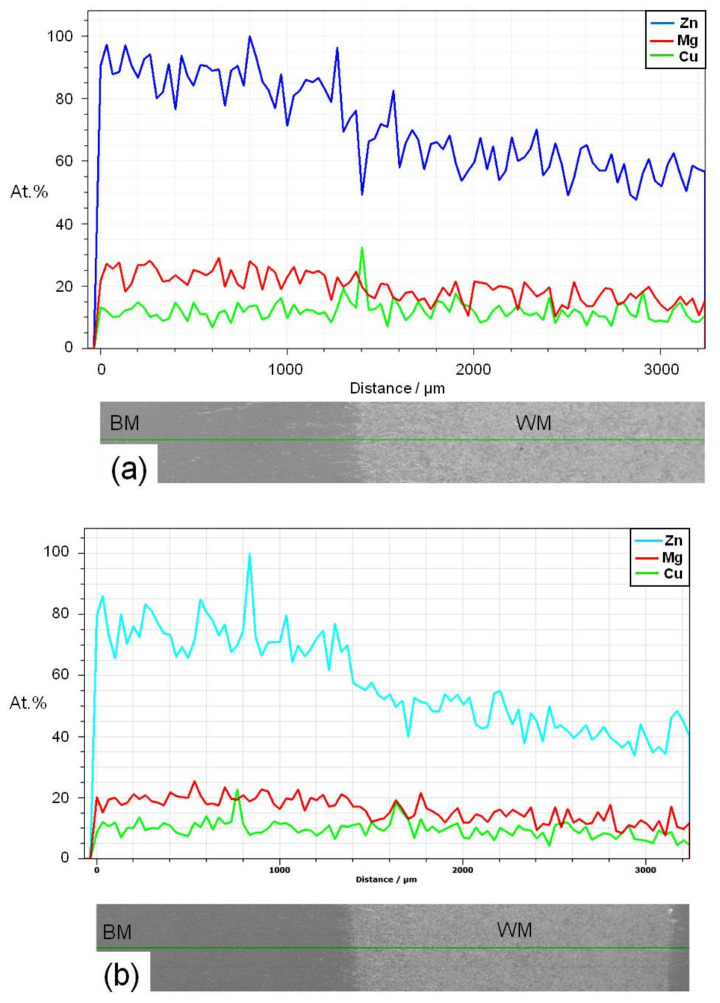
EDS line scan microanalysis showing the contents of Zn, Mg, and Cu elements along a 3 mm line crossing the fusion boundary of the low (**a**) and high (**b**) heat input welded joint.

The chemical composition of AA7075 aluminum alloy is balanced so that in the heat-treated state, it has a structure with a considerable amount of strengthening precipitates, which leads to a good combination of mechanical properties. Otherwise, this microstructure and mechanical properties are impacted by the fusion welding heat. The chemical composition of both the low and the high heat input welded joints was verified by EDS microanalysis for polished and etched cross-sections. In this regard, line scan microanalysis was carried out through the fusion boundary including BM, HAZ, and WM. An example of the typical composition profile of the low heat capacity welded joint is presented in Figure 12. The allocation of components is uneven, and their relative numbers have varied. The taller peak in this plot is a place where the stain overlies a precipitate in the HAZ/fusion line. In other words, this peak indicates that only a single precipitate is measured. It is obvious that the WM chemical composition is different from that of the BM. In comparison with the Cu profile that indicates similar Cu contents in both BM and WM, the high Zn and Mg contents of the base metal are remarkably decreased in the WM. This is due to the intense evaporation of Zn and Mg during welding. In other words, the allocation of alloying components and second-phase particles was considerably altered after welding, since the temperature with the weld pool exceeds both the vaporization temperature of the alloying elements and the dissolution temperature of the precipitates. A further decrease in WM chemical composition was reported for the high heat input joint due to its further evaporation of both Zn and Mg elements.

The results of EDS microanalysis are summarized in Table 4. There is an average of 0.85 wt % Zn, 0.5 wt % Mg, and 0.06 wt % Cu that has evaporated during welding. When we consider the steam pressure of the three elements, we see that the difference in pressure for Zn and Mg is only one order of magnitude, and between Zn/Mg and Cu, it is almost 12 orders of magnitude. From the steam pressure value, it can be inferred that Mg and Zn are evaporating more rapidly than Cu. The composition of the weld is still consistent with that of the AA7075 alloy, which is significant because much of the strength of the weld can be restored by natural aging at room temperature.

### 3.5. Tensile Properties of Welded Joints

**Figure 13:**
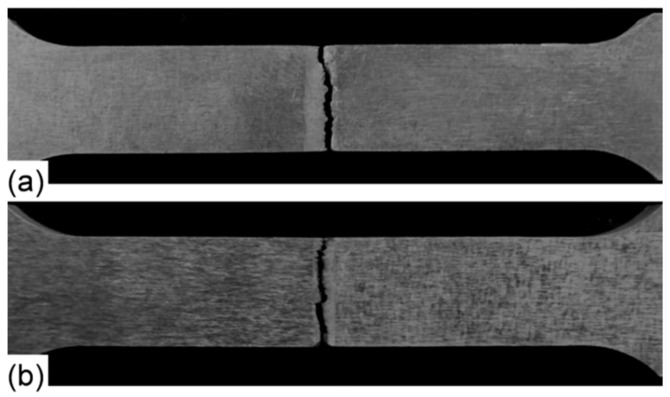
Photographs of tensile fractured specimens of the low (**a**) and high (**b**) heat input welds.

**Figure 14:**
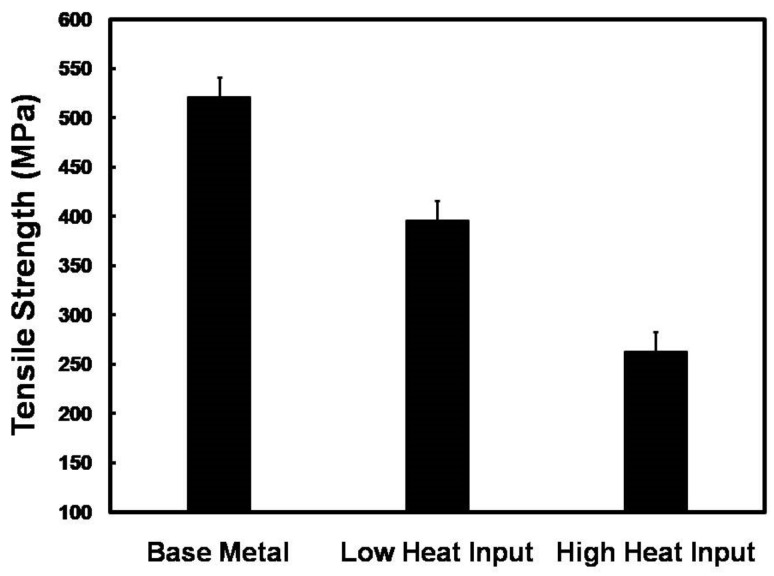
Tensile strength of the low and high heat input welds both with that of the BM.

Photographs of the tensile test and the destroyed samples of the low and high heat input welds are shown in Figure 13. Note that the front and back sides of the tensile-welded samples were processed and polished to obtain a plane surface in order to eliminate the effect of stress amplification. The fracture occurred in the WM of both the low (Figure 13a) and high (Figure 13b) heat input welded joints, which indicates a lower tensile strength and a lower plastic deformation capability of the WM of both joints as compared with the BM. Despite the presence of cracks, according to the results of fractographic studies, we see that the character of the fracture is ductile, the fracture process goes mainly along the grain boundaries and interdendritic spaces. The structure of the material in all zones of the welded joint is fine-grained. This neutralizes the presence of microcracks. This result corresponds quite well with the results of hardness measures where the lower hardness values were obtained in the WM (Figure 11). Deterioration in the mechanical properties of the welds is related to the deterioration of the WM microstructure including an elemental loss, coarse grain, and dissolution of strengthening precipitates, resulting in weaker grain boundary. Figure 14 demonstrated the tensile strength of the low and high heat input welds, with both that of the BM. The high heat input weld experienced the lower tensile strength (266 MPa) that is about half of that of the base metal (521 MPa). This considerable reduction in the tensile strength is attributed mainly to the remarkable degradation in WM microstructure that included an intense evaporation of alloying elements and dissolution of strengthening precipitates that in turn resulted in severe internal micro-porosity and micro-cracking. A much higher tensile strength of 396 MPa, which is 76% of that of the BM, was achieved for the low heat input weld. This remarkable recovery of the tensile strength is attributed mainly to a better WM quality due to a less microstructural degradation. The low heat input minimizes the fusion zone size, which means minimizing both the evaporation of the alloying elements and dissolution of the strengthening precipitates. In addition, the high cooling rate in this case minimizes both coarsening of the WM dendritic structure and intergranular segregation. In turn, these minimize the formation of micro-porosity and micro-cracking.

The mechanical properties of AA7075-T6 welded joints are remarkably affected by the excessive evaporation of alloying elements, dissolution of strengthening precipitates, and intergranular segregation as a function of the heat input. High heat input with its slow cooling rate caused a wider fusion zone with coarse dendritic structure, excessive loss of alloying elements, high dissolution degree of strengthening precipitates, and high degree of intergranular segregation. This eventually led to a significant deterioration of the welded joint mechanical properties. On the other hand, the lower heat input with its higher cooling rate caused a lower size of the fusion zone with a fine dendritic structure, limited loss of alloying elements, less dissolution of strengthening precipitates, and less interdendritic segregation. In turn, this resulted in higher hardness and tensile strength as well as lower mismatching of the low heat input welded joint.

**Figure 15:**
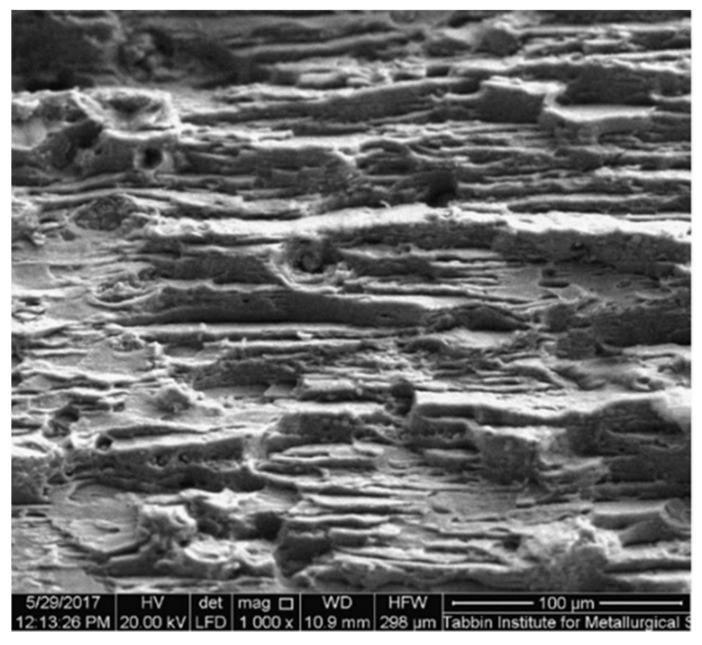
SEM photographs of the tensile fracture surface of the base metal.

**Figure 16:**
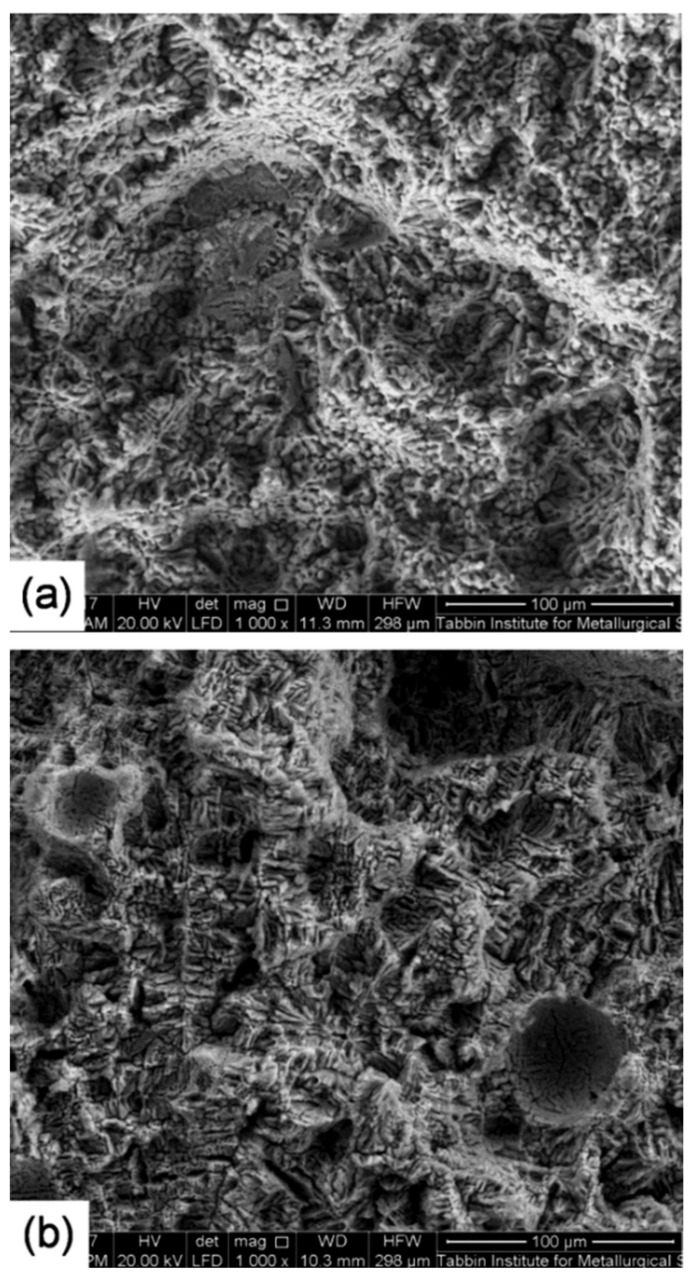
SEM photographs of the tensile fracture surface of specimens taken from the low heat input (**a**) and the high heat input (**b**) welded joints.

For further analysis of the break behavior, the break morphology was observed using a scanning electron microscope. SEM photos of the tensile breaking area of samples taken from the base of the metal as well as the low and high heat input welded joints are shown in Figure 15 and Figure 16, respectively. The BM fracture had larger and deeper pits, and there were noticeable rupture crests around the pits. It was also possible to clearly observe fragmented grains in the BM break. These grains represented a strengthening secondary phase that played a function in increasing the strength of the BM. In other words, the tensile fracture surface of the BM showed a ductile fracture appearance where dimple characteristics are clearly seen along the elongated grain structure, which is possibly caused by the tensile load parallel to the rolling direction (Figure 15). The fracture surface of the low heat input weld contains a large number of fine small-sized and shallow dimples (≈10 µm) as well as tearing ridges around the simples, so that this weld was also of ductile failure morphology (Figure 16a). However, on the surface of the weld, the depth and size of the pit were clearly less than on the BM fracture, indicating that the BM had greater extension than the weld. On the other hand, the fracture surface of the high heat input weld showed a brittle fracture appearance where cleavage-like features can be seen through the weld dendritic structure (Figure 16b). No hardening phase was detected in the fracture area of the weld, which was one of the reasons why the tensile strength of both weld specimens was worse than that of the BM. Another reason was that the rolled structure of the BM had a better strength than the cast structure of the weld specimens. It can be noticed that the fracture mode is a function of the level of internal welding defects as a function of the heat supplied. Compared to the ductile–brittle break surface at low thermal load, the higher degree of WM internal micro-pores and micro-cracking of the high heat input weld has resulted in brittle fracture mode. According to the results of studies of the chemical composition (Figure 10) and fracture analysis, we assume that the structure contains compounds based on copper and zinc.

The fusion zone size and microstructure, as a function of heat input, are considered as the main controlling factor in recovering both the hardness and tensile strength of the welded joints. A smaller fusion zone size having a fine dendritic structure, limited elemental loss, less dissolution of strengthening precipitates, and less intergranular segregation is of remarkable importance for recovering the hardness and tensile strength of the welds. This is feasible using the low heat input that restricted micro-pores, hot cracking, and intergranular segregation. The local hardness and overall tensile strength of the low heat input weld are 74% and 76% of those of the base metal, respectively. This result is much better than that of fusion arc welded joints where their achievable mechanical properties are less than 50% of those of the BM. The improved mechanical properties of the low heat input laser-welded joints make the weld more formable.

## 4. Conclusions

The objective of the current research work is to develop an in-depth understanding of the effect of high-power fiber laser on the mechanical properties of autogenous welds of AA7075-T6 sheets depending on the amount of heat input. In comparison with the high heat input, the low heat input caused a smaller fusion zone size with a fine dendritic structure, limited loss of alloying elements, less dissolution of strengthening precipitates, less intergranular segregation, as well as lower degree of residual tensile stress. Then, the WM soundness was improved due to a lower degree of porosity and hot cracking that in turn increased the local micro-hardness and total tensile strength of the low heat input-welded seam. The obtained local micro-hardness and overall tensile strength are 74% and 76% of those of the BM. These results are much better than those of fusion arc welded joints, which are lower than 50% of that of the BM. This improvement in mechanical properties results in a less mechanical dissimilarity that is of great significance for making the weld more formable.

As a new concept in this research, the local micro-hardness and the overall tensile properties of high-power fiber laser autogenous welded joints are dependent not only on the fusion zone size and microstructure but also on the degree of its mechanical mismatching as a function of the welding heat input. A smaller fusion zone dimension with lower degraded microstructure and less mechanical dissimilarity is of great significance for the attainment of better performance of high power fiber laser-welded joints. The implemented high-power fiber laser has enabled producing a low heat input welded joint using high welding speed, which is of remarkable importance for the minimizing fusion zone size, deterioration in structure, and mechanical mismatching. A high welding speed is desirable also in terms of the production rate. In other words, the recovering of mechanical properties of the high-strength AA7075 welded sheets is feasible using high-power fiber laser.

## Figures and Tables

**Table 1:** Chemical composition and mechanical properties of the used AA7075 aluminum plate.

Chemical Composition (wt %)								
Si	Fe	Cu	Mn	Mg	Cr	Zn	Ti	Al
0.14	0.23	1.37	0.16	2.51	0.21	5.56	0.03	balance
Mechanical Properties								
0.2% Proof stress (MPa)			Tensile strength (MPa)		Elongation (%)		Hardness (HV)	
453			521		8		189	

**Table 2:** Welding parameters used for laser beam welding.

Power (P) (kW)	Welding Speed (S) (m/min)	Defocusing Distance (Dd) (mm)	Top and Bottom Sides Shielding Gas/Flow Rate (L/min)
5.0–9.0	2.0–7.8	−10.0 to +10.0	Argon/13.0–25.0

**Table 3:** Welding parameters used for producing low and high heat input autogenous square butt-welded joints.

Power (P) (kW); Heat Input (kJ/m)	Welding Speed (S) (m/min)	Defocusing Distance (Dd) (mm)	Top and Bottom Sides Shielding Gas/Flow Rate (L/min)
9/(90 kJ/m)	6	−10.0	Argon/20
6/(180 kJ/m)	2	−10.0	Argon/20

**Table 4:** Amount of Zn, Mg, and Cu present in parent material and in the weld.

	BM (wt %)	WM (wt %)	Difference (wt %)	Vapor Pressure (mbar)	Tm (K)
Zn	5.56	4.71	0.85	7.53	693
Mg	2.51	2.01	0.5	0.49	923
Cu	1.37	1.31	0.06	3.24 × 10^−12^	1358

## Data Availability

The data presented in this study are available on request from the corresponding author.
